# Fucoidan Inhibits NLRP3 Inflammasome Activation by Enhancing p62/SQSTM1-Dependent Selective Autophagy to Alleviate Atherosclerosis

**DOI:** 10.1155/2020/3186306

**Published:** 2020-08-06

**Authors:** Yufei Cheng, Xudong Pan, Jing Wang, Xu Li, Shaonan Yang, Ruihua Yin, Aijun Ma, Xiaoyan Zhu

**Affiliations:** ^1^Department of Neurology, The Affiliated Hospital of Qingdao University, Shandong 266100, China; ^2^Institute of Cerebrovascular Diseases, The Affiliated Hospital of Qingdao University, Shandong 266100, China; ^3^Department of Critical Care Medicine, The Affiliated Hospital of Qingdao University, Shandong 266100, China

## Abstract

NOD-like receptor family pyrin domain containing 3 (NLRP3) inflammasome activation contributes to the progression of atherosclerosis, and autophagy inhibits inflammasome activation by targeting macrophages. We investigated whether fucoidan, a marine sulfated polysaccharide derived from brown seaweeds, could reduce NLRP3 inflammasome activation by enhancing sequestosome 1 (p62/SQSTM1)-dependent selective autophagy to alleviate atherosclerosis in high-fat-fed ApoE-/- mice with partial carotid ligation and differentiated THP-1 cells incubated with oxidized low-density lipoprotein (oxLDL). Fucoidan significantly ameliorated lipid accumulation, attenuated progression of carotid atherosclerotic plaques, deregulated the expression of NLRP3 inflammasome, autophagy receptor p62, and upregulated microtubule-associated protein light chain 3 (LC3)-II/I levels. Transmission electron microscopy and GFP-RFP-LC3 lentivirus transfection further demonstrated that fucoidan could activate autophagy. Mechanistically, fucoidan remarkably inhibited NLRP3 inflammasome activation, which was mostly dependent on autophagy. The inhibitory effects of fucoidan on NLRP3 inflammasome were enhanced by autophagy activator rapamycin (Rapa) and alleviated by autophagy inhibitor 3-methyladenine (3-MA). Fucoidan promoted the colocalization of NLRP3 and p62. Knockdown of p62 and ATG5 by small interfering RNA significantly reduced the inhibitory effects of fucoidan treatment on NLRP3 inflammasome. The data suggest that fucoidan can inhibit NLRP3 inflammasome activation by enhancing p62/SQSTM1-dependent selective autophagy to alleviate atherosclerosis.

## 1. Introduction

Ischemic stroke is a destructive cerebrovascular disease worldwide, with a high rate of death, disability, and recurrence [1]. The main pathogenesis mechanism of ischemic stroke is carotid atherosclerosis, which is recognized as a chronic vascular inflammatory disease [2]. The unbalanced activation of plaque resident macrophages and the overexpressed inflammatory cytokines interleukin-1*β* (IL-1*β*) contribute to the formation and rupture of atherosclerotic plaques [3]. Among the multitudinous pathways that regulate the maturation and secretion of IL-1*β*, the classical NOD-like receptor family pyrin domain containing 3 (NLRP3) inflammasome-dependent pathway is of great significance [4].

During the development of atherosclerosis, the NLRP3 inflammasome is inappropriately activated by cholesterol crystals, oxidized low-density lipoprotein (oxLDL), and abnormal hemodynamics, which results in massive inflammation [5]. The NLRP3 inflammasome is a cytosolic protein complex consisting of the NLRP3, apoptosis-associated speck-like protein (ASC), and protease caspase-1, which can cleave pro-IL-1*β* into mature IL-1*β*, thus aggravating the inflammatory response in atherosclerosis [6]. A genetic deficiency of NLRP3, ASC, or caspase-1 results in the low production of IL-1*β* and stabilizes atherosclerotic plaques in ApoE-/- mice [7–9]. Emerging evidence has suggested that autophagy is the essential process in controlling NLRP3 inflammasome activation [10].

Autophagy is a highly conserved cellular degradation process that can traffic damaged organelles or misfolded proteins to lysosomes for clearance [11]. We previously demonstrated that the autophagy inducer Rapa plays a protective role in the progression of atherosclerotic plaques [12]. Several basic examinations also revealed that mice with a macrophage-specific deletion of the essential autophagy gene ATG5 developed plaques with increased oxidative stress and exhibited enhanced plaque necrosis [10, 13]. It has been proposed that autophagic adaptor protein p62 participates in selective autophagy to directly engulf and eliminate ubiquitinated NLRP3 and ASC by binding with LC3 [14, 15]. Furthermore, p62 ablation contributes to lipid deposition, increased secretion of IL-1*β*, and increased formation of plaques in ApoE-/- mice [16]. These observations prompt us to speculate that the effective inhibition of the NLRP3 inflammasome pathway via autophagy is essential to stabilize atherosclerotic plaques and prevent the development of atherosclerosis.

Fucoidan, a natural polysaccharide mainly made of fucose and sulfate, is abundant in brown seaweeds and widely utilized in the cosmetic and food industries [17]. Fucoidan has also long been considered an attractive compound as a drug in traditional Chinese medicine [18]. Recently, various pharmacological properties with the potential medicinal value, such as antioxidant, anti-inflammatory, antiproliferative, and anticoagulant activities have been described in fucoidan [19–22]. A study reported that fucoidan significantly reduced atherosclerotic plaques in the aortic arch of spontaneously hyperlipidemic (Apo*^shl^*) mice [23]. However, the exact effects and biochemical mechanisms of fucoidan in terms of inflammation and atherosclerosis are unclear. No reports have addressed fucoidan-mediated regulation of the NLRP3 inflammasome and autophagy in atherosclerosis.

In the present study, we used ApoE-/- mice, which exhibit the development of more severe atherosclerotic lesions than Apo*^shl^* mice, to generate a widely used vulnerable carotid atherosclerotic plaque model involving the ligation of the carotid artery and a high-fat, high-cholesterol diet [24, 25]. We analyzed whether fucoidan can stabilize the vulnerable atherosclerotic plaques and whether this stabilization is due to regulation of the NLRP3 inflammasome and autophagy. At the same time, a foam cell model was established *in vitro* to investigate the underlying mechanism.

## 2. Materials and Methods

### 2.1. Drugs and Reagents

Fucoidan extracted from the brown seaweed *Fucus vesiculosus* by a modified method was purchased from Sigma-Aldrich (St. Louis, MO, USA; #F5631). Fucoidan was dissolved in distilled water, stirred at 25°C for 30 min, filtered through a 0.22 *μ*m pore size filter (Millipore, Billerica, MA, USA), and stored at -20°C until use [26]. oxLDL made by oxidizing human LDL using Cu_2_SO_4_ (oxidant) in PBS at 37°C for 20 h was purchased from Yiyuan Biotech (Guangzhou, China; Cat. No. YB-002). All cell culture consumables were purchased from the Corning Company (New York, NY, USA). The following reagents and their suppliers were also acquired: fetal bovine serum and RPMI 1640 medium (Biological Industries, Beit HaEmek, Israel), penicillin-streptomycin (Hyclone, Logan, UT, USA), phorbol-12-myristate-13-acetate (PMA), BafA1, 3-MA, and rapamycin (MCE Company, Monmouth Junction, NJ, USA), and Lipofectamine™ 2000 reagent (Invitrogen, Carlsbad, CA, USA).

### 2.2. Animal Models and Ethics Statements

All experiment procedures followed the ARRIVE guidelines for the reporting of experiments involving animals [27]. The animal experimental protocol was reviewed and approved by the ethics committee of Qingdao University (QUMC 2018-08). Thirty-six (C57BL/6 background) 6-week-old-male ApoE-/- mice (Huafukang Biotechnology Company, Beijing, China) were housed in the animal center at standard room temperature and a 12-h light/dark cycle conditions, with three to four mice per cage. The ApoE-/- mice were randomly divided into the following three groups (*n* = 12 per group): control group, model group, and fucoidan group. All mice were fed with normal food 2 weeks before surgery. At 8 weeks of age, ApoE-/- mice in the control group were fed with normal food without surgery. In the other two groups, silica gel rings were inserted into the right common carotid artery, and the mice were fed a high-fat, high-cholesterol diet (0.25% cholesterol, 15% cocoa butter, and basic diet; License No.: SCXK-Beijing-2014-0008, China) to induce carotid atherosclerotic lesion formation. At 12 weeks of age, the mice in the fucoidan group each received an intraperitoneal injection of fucoidan (60 mg kg^−1^day^−1^), and the mice in the model group were injected intraperitoneally with physiological saline once per day [28, 29]. At 16 weeks of age, all mice were anesthetized using isoflurane and sacrificed by inner canthus artery exsanguination while still anesthetized [30].

### 2.3. Lipid Analysis

Blood was withdrawn from the inner canthus artery and centrifuged at 1000 × *g*. Serum biochemical analyses including total cholesterol (TC), triglyceride (TG), and low-density lipoprotein cholesterol (LDL-c) were performed in the clinical laboratory of the Affiliated Hospital of Qingdao University.

### 2.4. Histology

The right common carotid arteries of all groups were rapidly removed and fixed in 4% paraformaldehyde, embedded in optimum cutting temperature compound (Sakura Finetek USA, Torrance, CA, USA), and stored at -80°C until use. Blood vessel tissue sections approximately 7 *μ*m in thickness were selectively stained with Oil Red O and hematoxylin-eosin (HE) for the observation of carotid atherosclerotic plaque formation using a microscope (Olympus, Tokyo, Japan).

### 2.5. Immunohistochemical Staining

After the carotid arteries were dried and dewaxed using dimethyl benzene, arteries were rehydrated with graded ethanol solutions. The endogenous peroxidase activity was blocked using 3% hydrogen peroxide for 20 min. After treatment with fetal bovine serum, the arteries were incubated overnight at 4°C with anti-NLRP3 antibody (Novus, Littleton, Co, USA) and anti-p62 antibody (Abcam, Cambridge, MA, USA). The samples were then incubated with a secondary antibody for 1 h at room temperature.

### 2.6. Cell Culture

THP-1 human monocytic leukemia cell line was obtained from Shanghai Institutes for Biological Sciences (Shanghai, China). Cells were cultured in RPMI 1640 medium containing 10% fetal bovine serum supplemented with 100 nM penicillin-streptomycin at 37°C and 5% CO_2_ in cell incubators. For experiments, logarithmic growth phase THP-1 cells (4 × 10^5^ cells mL^−1^) were differentiated into macrophages by treatment with 100 ng mL^−1^ of PMA for 48 h in dishes or wells. Subsequently, macrophages were transformed into foam cells treated with 80 *μ*g mL^−1^ oxLDL for 36 h. For experiments involving autophagy modulation, macrophages were pretreated with bafA1, rapamycin (25 nM), and 3-MA (5 mM) for 2 h and subsequently treated with fucoidan (300 *μ*g mL^−1^).

### 2.7. Transfection

The p62 and control siRNAs were purchased from Cell Signaling Technology (Danvers, MA, USA). Red fluorescent protein-green fluorescent protein-LC3 (RFP-GFP-LC3) adenoviral vectors, ATG5 siRNA, and control siRNA were designed and synthesized by Jikai (Guangzhou, China). THP-1 cells were transfected according to the manufacturer's protocol. Autophagic flux observation and mounting were performed with a fluorescence microscope (Olympus).

### 2.8. Analysis of Cell Viability

The 3-(4, 5-dimethylthiazol-2-yl)-2, 5-diphenyltetrazolium bromide (MTT) assay was used to analyze cell viability (Solarbio, Beijing, China). Briefly, THP-1 cells were plated in 96-well plates and differentiated into macrophages. After exposure to fucoidan and oxLDL, 20 *μ*L of MTT working solution was added to each well of the plate, and incubation was continued for 4 h at 37°C. The solution was removed, and 150 *μ*L of dimethyl sulfoxide (DMSO; Solarbio, Beijing, China) was added to each well for 30 min to dissolve the insoluble formazan that had accumulated from the reduction of MTT by viable cells. The plates were detected at 570 nm using a microplate reader (Bio-Rad, Hercules, CA, USA).

### 2.9. Oil Red O Staining

The foam cells were incubated with or without fucoidan for 36 h. After being washed three times with PBS, the cells were fixed with 4% paraformaldehyde for 25 min. Oil Red O solution was added and incubated for 30 min at 37°C. After extensive washing with PBS, the cells were immediately photographed using a microscope (Olympus).

### 2.10. Western Blot Analysis

All proteins extracted from carotid arteries or cells were separated by SDS-PAGE and transferred to polyvinylidene difluoride (PVDF) membranes (Millipore, Vimodrone, Milan, Italy). After blocking with 5% skim milk in PBS or 4% bovine serum albumin for 2 h at room temperature, the membranes were incubated with appropriate primary antibodies against NLRP3 (#15101, 1 : 800; Cell Signaling Technology, Danvers, MA, USA), ASC (#67824, 1 : 1000; Cell Signaling Technology), caspase-1 (ab1872, 1 : 1000; Abcam), IL-1*β* (ab9787, 1 : 1000; Abcam), LC3B (ab192890, 1 : 1000; Abcam), GAPDH (10494-1-AP, 1 : 5000; Proteintech, Rosemont, IL, USA), p62 (18420-1-AP, 1 : 1000; Proteintech), or ATG5 (#12994, 1 : 1000; Cell Signaling Technology) at 4°C overnight. The membranes were then incubated with horseradish peroxidase (HRP)-conjugated antirabbit or antimouse secondary antibody (1 : 10,000 dilution) for 1 h. Bands were visualized using an enhanced chemiluminescence (ECL) detection kit (Millipore Co, Billerica, MA, USA). The relative protein quantity was measured using ImageJ software (NIH, Bethesda, MD, USA).

### 2.11. Detection of IL-1*β* by Enzyme-Linked Immunosorbent Assay (ELISA)

The IL-1*β* ELISA kit was obtained from eBioscience (San Diego, CA, USA) and was used according to the manufacturer's protocol. Serum samples from ApoE-/- mice and cell culture medium were collected, centrifuged at 1000 g for 20 min, and IL-1*β* levels were assessed. The optical density of the peroxidase product was read at 450 nm. According to the standard curve, the concentration of IL-1*β* in each sample was measured.

### 2.12. Transmission Electron Microscopy (TEM)

The tissue or cells were fixed with 4% paraformaldehyde, postfixed in 1% osmium tetroxide, dehydrated in a graded ethanol series, infiltrated with propylene oxide, embedded in epoxy resins, and sectioned. After double staining with uranyl acetate and lead citrate, ultrathin sections were examined using a model HT-7700 transmission electron microscope (Hitachi, Tokyo, Japan).

### 2.13. Immunofluorescence

Analysis of colocalization between NLRP3 and LC3 in cells was performed using double immunohistochemistry staining. Briefly, after different drug treatments, the cells were fixed with 4% paraformaldehyde for 30 min and air-dried. Fixed cells were treated with 0.5% Triton X-100 for 10 min at room temperature and then blocked with goat serum 1 h. Subsequently, a solution containing diluted rabbit anti-NLRP3 antibody and mouse anti-p62 were added to the cells and incubated at 4°C overnight. Subsequently, Alexa Fluor 488 donkey antirabbit IgG and Alexa Fluor 555 donkey antimouse IgG were used as secondary antibodies (Invitrogen) in the dark for 1 h. After washing with PBS, the nuclei were stained by 4′, 6-diamidino-2-pheny-lindole (DAPI; Beyotime, Beijing, China) for 20 min. The images were examined using a fluorescence microscope (Olympus).

### 2.14. Statistical Analysis

The statistical analyses were performed with SPSS 19.0 software (IBM, Armonk, NY, USA). Continuous data are expressed as the mean ± SEM. Statistical significance among several groups was determined using one-way analysis of variance with Dunnett's post hoc test (ANOVA). A value of *p* < 0.05 was considered statistically significant.

## 3. Results

### 3.1. Fucoidan Reduces Lipid Levels and Ameliorates the Formation of Unstable Carotid Atherosclerotic Plaques in ApoE-/- Mice

**Figure 1:**
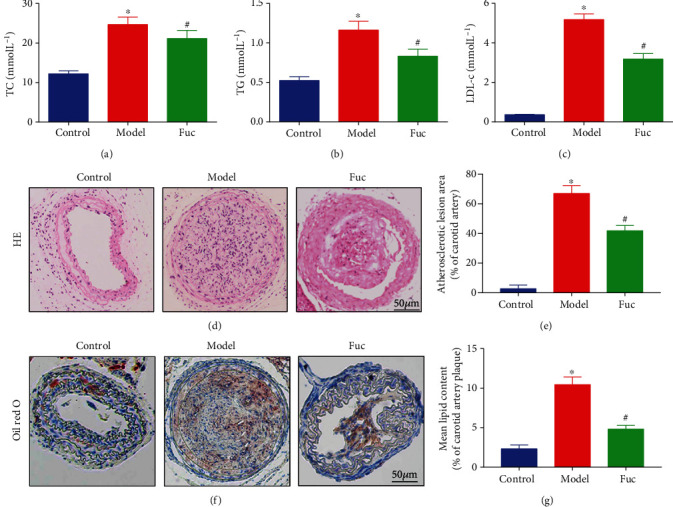
Fucoidan reduces lipid levels and alleviates carotid atherosclerotic plaques progression. (a–c) Plasma lipid levels of total cholesterol, triglyceride, and LDL cholesterol in ApoE-/- mice. (d, f) Effects of fucoidan on carotid atherosclerotic plaques stained with HE and Oil Red O. (e, g) Summarized data of the lesion area and lipid content of carotid arteries. Scale bar = 50 *μ*m in each panel. Data are shown as the mean ± SE (*n* = 12). ^∗^*p* < 0.05 vs. control; ^#^*p* < 0.05 vs. model.

We first investigated the impact of fucoidan on lipid metabolism, which is the main characteristic of atherosclerosis. As shown in Figures 1(a)–1(c), serum TC, TG and LDL cholesterol, and HDL cholesterol levels were obviously increased in the model group. In contrast, the fucoidan showed obviously decreased serum TC level (fucoidan group vs. model group: 19.85 ± 2.23 mmol L^−1^ vs. 26.68 ± 1.42 mmol L^−1^, *p* < 0.05), TG level (fucoidan group vs. model group: 0.68 ± 0.15mmol L^−1^ vs. 1.46 ± 0.19 mmol L^−1^, *p* < 0.05), and LDL cholesterol level (fucoidan group vs. model group: 3.05 ± 0.42 mmol L^−1^ vs. 5.57 ± 0.63 mmol L^−1^, *p* < 0.05).

To explore the potential effects of fucoidan on the formation of carotid atherosclerotic plaques in ApoE-/- mice, the carotid artery on the cannulated side in mice was stained with HE and Oil Red O. Compared with the control group, the model group showed obvious plaque formation, thrombosis, and lipid-laden foam cell infiltration. However, the formation of carotid atherosclerotic plaques and lipid disposition were significantly alleviated in the fucoidan group (Figures 1(d)–1(g)).

### 3.2. Fucoidan Inhibits the NLRP3 Inflammasome Activity in ApoE-/- Mice

**Figure 2:**
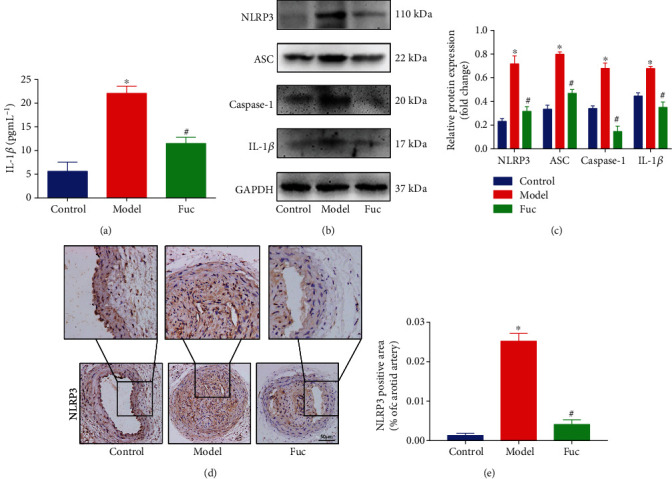
Fucoidan inhibits NLRP3 inflammasome activity in ApoE-/- mice. (a) The plasma levels of IL-1*β* in ApoE-/- mice were measured by ELISA. (b, c) Representative western blot analysis of the protein expression levels of NLRP3, ASC, caspase-1, and IL-1*β* in right carotid arteries. All data are representative of three independent experiments. (d, e) Immunohistochemical analysis of NLRP3 in carotid atherosclerotic plaques. Scale bar = 50 *μ*m. Data are presented as mean ± SE (*n* = 12). ^∗^*p* < 0.05 vs. control; ^#^*p* < 0.05 vs. model.

To explore the effect of fucoidan on NLRP3 inflammasome and IL-1*β* production in atherosclerosis, we examined the serum secretion of IL-1*β* using ELISA. As shown in Figure 2(a), the elevated levels of IL-1*β* in the model group were significantly decreased after fucoidan treatment. Western blotting was used to detect NLRP3 inflammasome component and IL-1*β* production in the carotid artery of mice. The protein levels significantly increased in the model group compared with those in the control group. However, treatment with fucoidan effectively decreased the levels of NLRP3, ASC, and caspase-1 compared with the control and model groups (Figure 2(b)). Consistent with these findings, immunohistochemistry analysis showed increased NLRP3 expression in the intima and necrotic core where macrophages were found in the model group. The NLRP3 level was markedly lower in the fucoidan group (Figures 2(d) and 2(e)). These findings revealed that fucoidan inhibited NLRP3 inflammasome activity and IL-1*β* production.

### 3.3. Fucoidan Enhances Autophagy in ApoE-/- Mice

**Figure 3:**
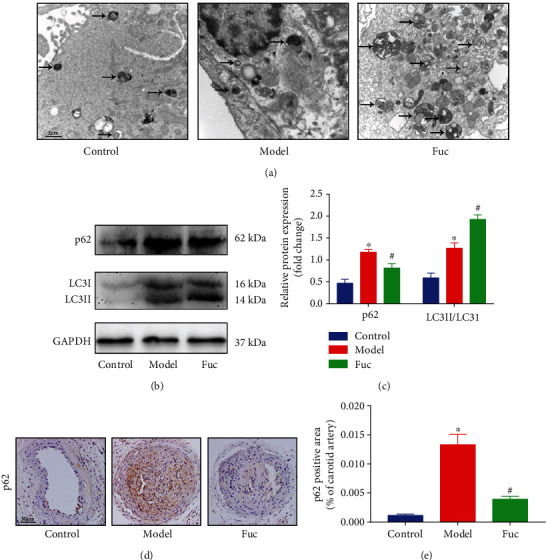
Fucoidan activates autophagy in ApoE-/- mice. (a) Detection of autophagosomes in unstable carotid atherosclerotic plaques by TEM. Scale bar = 2 *μ*m. (b, c) Representative western blot analysis of the protein expression levels of p62 and LC3 in the carotid arteries. All data are representative of three independent experiments. (d, e) Immunohistochemical analysis of p62 in carotid atherosclerotic plaques. Autophagosomes were indicated by arrows. Scale bar = 50 *μ*m. Data are shown as the mean ± SE (*n* = 12). ^∗^*p* < 0.05 vs. control; ^#^*p* < 0.05 vs. model.

TEM was used to detect autophagosomes and autolysosomes in the carotid arteries. The number of autophagosomes in the carotid arteries of ApoE-/- mice increased significantly after fucoidan treatment (Figure 3(a)). The expression of autophagy markers LC3 and p62 was examined by western blot. The level of LC3II/LC3I in the model group was not obviously decreased compared with that in the control group, but the expression of p62 was significantly increased, indicating that autophagic flux was inhibited. Fucoidan treatment significantly increased the LC3II/LC3I level and decreased the p62 level to a normal extent, indicating enhanced autophagic flux (s 3(b) and 3(c)). Consistently, immunohistochemistry analysis showed the same results (Figure 3(d)). Taken together, these results suggested that fucoidan could activate autophagy in ApoE-/- mice.

### 3.4. Fucoidan Reduces Lipid Accumulation in OxLDL-Treated Macrophages

**Figure 4:**
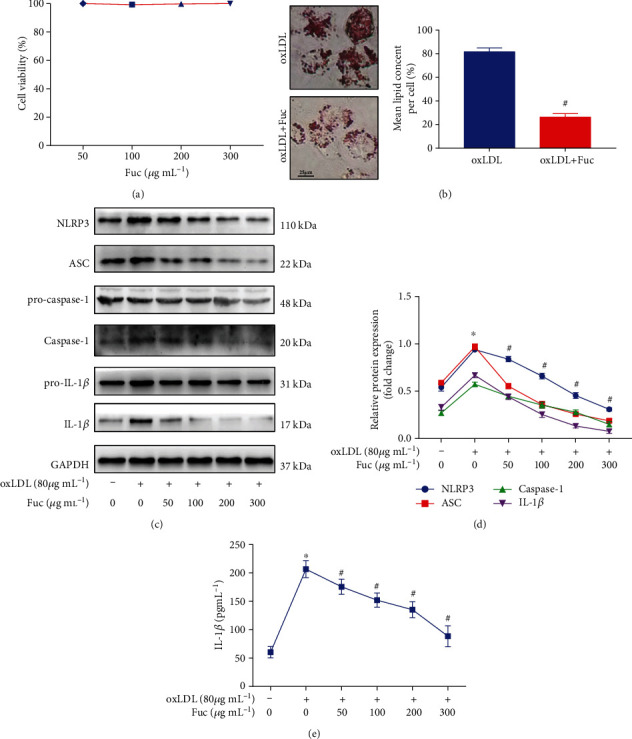
Fucoidan reduces lipid accumulation and inhibits NLRP3 inflammasome activation in oxLDL-treated macrophages. (a) MTT was used to measure the cell viability in foam cells incubated with different doses of fucoidan for 36 h. (b) Intracellular lipid accumulation was assessed by Oil Red O staining. Scale bars = 25 *μ*m. (c, d) Representative western blot analysis of the protein expression levels of NLRP3, ASC, pro-caspase-1, caspase-1, pro-IL-1*β*, and IL-1*β*. All data are representative of three independent experiments. (e) The levels of IL-1*β* in the culture medium were measured by ELISA. Data are shown as the mean ± SE. ^∗^*p* < 0.05 vs. the untreated group; ^#^*p* < 0.05 vs. the oxLDL-treated group.

To further investigate the specific molecular mechanism of fucoidan in atherosclerosis, we established a foam cell model by inducing foam cell formation in macrophages treated with oxLDL. After foam cells were incubated with different concentrations of fucoidan for 36 h, cytotoxicity was detected using the MTT assay, and lipid droplets were observed with Oil Red O staining. Fucoidan treatment did not affect the viability of the foam cells (Figure 4(a)). The staining results revealed that the oxLDL treatment group displayed a great number of lipid droplets, which accounted for more than 50% of the cell volume. However, compared with oxLDL treatment, fucoidan treatment reduced the intracellular lipid droplet content (Figure 4(b)).

### 3.5. Fucoidan Inhibits OxLDL-Induced Inflammasome Activation in a Dose-Dependent Manner

Previous studies have demonstrated that oxLDL induces an inflammatory response and activates the NLRP3 inflammasome. Presently, fucoidan significantly inhibited the oxLDL-induced NLRP3 inflammasome-associated protein expression in a dose-dependent manner (Figures 4(c) and 4(d)). We further used ELISA to assess cytokine IL-1*β* secretion in the cell culture medium. OxLDL could induce the secretion of IL-1*β* from cells, and fucoidan inhibited the oxLDL-induced secretion of IL-1*β* (Figure 4(e)).

### 3.6. Fucoidan Alleviates the Impairment of Autophagic Flux Induced by OxLDL in a Dose-Dependent Manner

**Figure 5:**
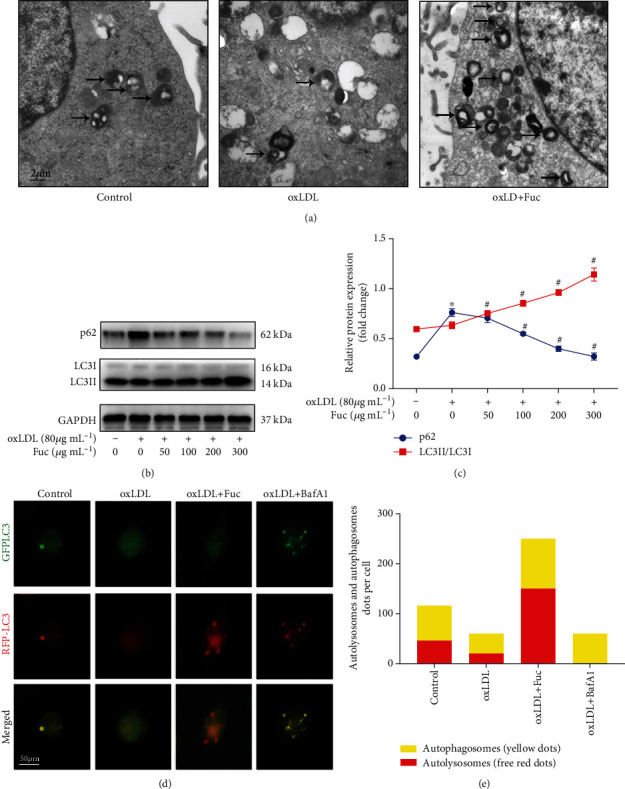
Fucoidan enhances the impaired autophagy flux induced by oxLDL. (a) Autophagosomes in THP-1 cells detected by TEM. Autophagosomes are indicated by arrows. Scale bars = 2 *μ*m. (b, c) Representative western blot analysis of the protein expression levels of LC3II/LC3I and p62. All data are representative of three independent experiments. (d) Fluorescence microscopy images of GFP-RFP-LC3 adenovirus transfected THP-1 cells treated with oxLDL, oxLDL+ fucoidan, or 50 nM baf A1 for 36 h. Scale bars = 50 *μ*m. (e) The number of autolysosomes (red puncta) per cell was counted. Data are shown as the mean ± SE. ^∗^*p* < 0.05 vs. the untreated group; ^#^*p* < 0.05 vs. the oxLDL-treated group.

OxLDL inhibits autophagy in macrophages and promotes the development of atherosclerosis [31]. In addition, autophagy induction results in NLRP3 inflammasome degradation and reduced IL-1*β* secretion. Hence, we hypothesized that fucoidan could rescue autophagic flux in foam cells. TEM showed that treatment with fucoidan (300 *μ*g mL^−1^) increased the formation of autophagosomes (Figure 5(a)). As shown in Figures 5(b) and 5(c), fucoidan significantly upregulated LC3II/LC3I and downregulated p62 expression in a dose-dependent manner. To further study fucoidan-induced autophagy, we used GFP-RFP-LC3 adenovirus transfection to observe autophagic flux. Because GFP is unstable in acidic conditions, weakening of the GFP signal indicates that lysosomes fuse with autophagosomes to form autolysosomes, resulting in quenching of GFP fluorescence. As demonstrated in Figures 5(d) and 5(e), the abundant formation of red puncta indicating autolysosomes was observed in the fucoidan-treated (300 *μ*g mL^−1^) group, and few red puncta were observed in the bafA1 group. These results suggested that fucoidan treatment enhanced the impaired autophagy flux induced by oxLDL.

### 3.7. Fucoidan Mediates P62-Dependent Selective Autophagy of NLRP3 Inflammasome

**Figure 6:**
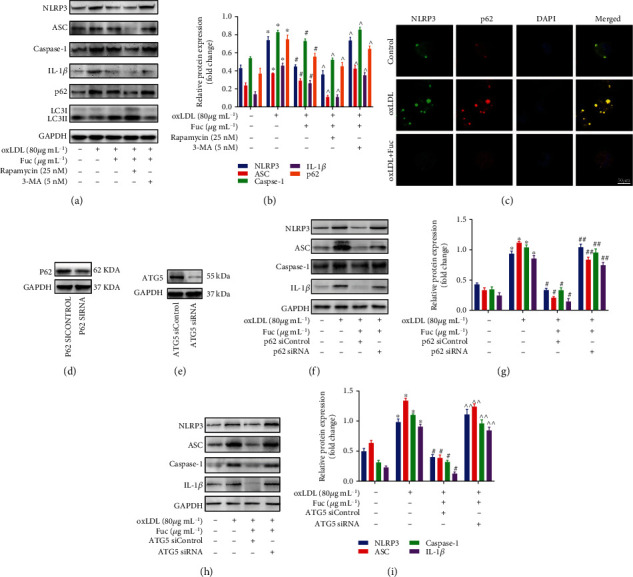
Fucoidan mediates p62-dependent selective autophagy of NLRP3 inflammasome. (a, b) Representative western blot analysis of the protein expression levels of NLRP3 inflammasome-associated proteins, LC3II/LC3I, and p62. All data are representative of three independent experiments. (c) Fluorescence microscopy revealing the accumulation of NLRP3 and p62. Green indicates NLRP3 staining, red indicates p62 staining, and yellow indicates colocalization of NLRP3 and p62. Scale bar = 50 *μ*m. (d) P62 protein expression following transfection with the control or p62 siRNA for 72 h. (e) ATG5 protein expression following transfection with the control or ATG5 siRNA for 72 h. (f–i) Representative western blot analysis of the protein expression levels of NLRP3, ASC, caspase-1, and IL-1*β*. All data are representative of three independent experiments. Data are shown as the mean ± SE. ^∗^*p* < 0.05 vs. the untreated group; ^#^*p* < 0.05 vs. the oxLDL-treated group; ^^^*p* < 0.05 vs. the oxLDL + Fuc-treated group; ^##^*p* < 0.05 vs. the oxLDL+ Fuc + p62 siControl-treated group. ^^^^*p* < 0.05 vs. the oxLDL+ Fuc + ATG5 siControl-treated group.

A large body of evidence has shown that autophagy links the inflammasome [10]. To explore the relationship between autophagy inhibition and NLRP3 inflammasome activation by fucoidan, we used the autophagy inducer Rapa and the autophagy inhibitor 3-MA. As shown in Figure 6(a), the protein levels of NLRP3, ASC, caspase-1, and IL-1*β* were markedly increased in 3-MA pretreated macrophages exposed to fucoidan (300 *μ*g mL^−1^), indicating that the anti-inflammatory effect of fucoidan was reversed. In contrast, pretreatment of the macrophages with rapamycin led to lower IL-1*β* secretion and NLRP3 inflammasome expression (Figure 6(a)). These data indicate that fucoidan can inhibit inflammasome activity by activating autophagy.

Immunostaining showed that fucoidan-induced autophagy increased the colocalization of NLRP3 and p62 (Figure 6(c)). The autophagic adaptor p62 can bind and deliver substrates to the autophagosome for degradation [13]. To further verify the role of p62 in fucoidan-mediated regulation of the NLRP3 inflammasome, macrophages were transfected with p62-siRNA to silence p62 expression (Figure 6(d)). The expression levels of NLRP3, ASC, caspase-1, and IL-1*β* in p62-siRNA-treated cells were higher than in siRNA negative control cells after fucoidan treatment (Figures 6(f) and 6(g)). Next, we silenced autophagy using ATG5 siRNA (Figure 6(e)). The expression levels of NLRP3, ASC, caspase-1, and IL-1*β* were enhanced in fucoidan-treated macrophages (Figures 6(h) and 6(i)). The collective results indicated that fucoidan-induced autophagy could selectively degrade the NLRP3 inflammasome via p62.

## 4. Discussion

Although it was shown that atherosclerosis in the ApoE-/- mice was prevented by fucoidan in a few reports, the molecule mechanism of the protection remains unknown [32]. The present study is the first evidence that fucoidan inhibits NLRP3 inflammasome activation by enhancing p62/SQSTM1-dependent selective autophagy to alleviate atherosclerosis.

We established a carotid vulnerable atherosclerotic plaque model in ApoE-/- mice and confirmed that exposure to fucoidan significantly ameliorated lipid accumulation, delayed the development of carotid atherosclerotic plaques, inhibited the NLRP3 inflammasome, and activated autophagy. To further explore the mechanism underlying the relationship between NLRP3 and autophagy after using fucoidan, oxLDL was used to induce macrophages into foam cells. We found that fucoidan attenuated foam cell formation, suppressed the NLRP3 inflammasome, and enhanced autophagy. Moreover, the fucoidan-mediated antiatherosclerotic effects were mediated by p62-dependent autophagy, which selectively degraded the NLRP3 inflammasome.

In a groundbreaking paper, Gerrity et al. identified macrophages as the main component of the atherosclerotic plaque [33]. Macrophages, derived from circulating monocytes, produce proinflammatory cytokines, participate in lipid retention and vascular cell remodeling, and express Toll-like receptors that connect the innate and adaptive immune response during atherosclerosis [34]. Recent studies have shown that fucoidan exerts anti-inflammatory effects by decreasing the secretion of IL-1*β*, a proinflammatory cytokine secreted by macrophages [22, 35]. IL-1*β* production is a tightly controlled process that typically requires NLRP3 inflammasome pathway activation [6]. We previously described that the activation of the NLRP3 inflammasome contributes to the development of atherosclerosis in high-fat-fed ApoE-/- mice with partial carotid ligation and differentiated THP-1 cells incubated with oxLDL [12, 36]. Inhibition of the NLRP3 inflammasome results in decreased inflammation and reduced atherosclerosis [37]. However, to the best of our knowledge, the fucoidan-mediated regulation of NLRP3 inflammasome has not previously been reported in atherosclerosis. The present study is the first description that fucoidan, one of the most abundant extracts obtained from brown seaweed, can reverse the activation of the NLRP3 inflammasome that accelerates the development of atherosclerosis. The findings suggest that the antiatherosclerotic mechanism could be associated with the decrease of the NLRP3 inflammasome. The same results were obtained in foam cells after fucoidan treatment. Therefore, the NLRP3 inflammasome might be the key regulator accounting for the protective effects of fucoidan on atherosclerosis.

Accumulating evidence suggests that autophagy negatively regulates the activation of the NLRP3 inflammasome to maintain homeostasis [38]. For example, deficiency of the autophagy gene Atg16L1 leads to the secretion of IL-1*β* and the activation of the NLRP3 inflammasome [39]. Another study reported that once autophagy activators were used, NLRP3 inflammasome activity was significantly inhibited and IL-1*β* secretion was significantly reduced [40]. Importantly, autophagy has been associated with the fate of macrophage-derived foam cells and atherosclerotic plaques [41]. It is worth mentioning that fucoidan can induce autophagy through reduced phosphorylation of key components of the phosphoinositide 3-kinase/Akt/mammalian target of rapamycin pathway in cancer cells [42]. Based on these data, we speculate that fucoidan can activate autophagy, resulting in the inhibition of NLRP3 inflammasome activity in atherosclerosis. First, we used TEM to investigate the effect of fucoidan on autophagy. As expected, many autophagic vesicles and lysosomes were apparent in the fucoidan group. P62/SQSTM-1, which binds to LC3, is often used as a marker of autophagic flux because its accumulation is indicative of a blockage in autophagy [43]. In this study, we found that p62 expression was significantly increased and autophagy flux was blocked in the model group and foam cells, while fucoidan treatment reversed the effect and promoted the transition of LC3 from type I to type II. To further evaluate the autophagic flux, we used lentivirus to transfect GFP-RFP-LC3 into cells to detect autophagic flux. After using fucoidan, the number of red spots increased, indicating an increase in autolysosomes. In contrast, when autophagy was blocked with the late autophagy inhibitor bafA1, a marked reduction in autolysosomes was observed. These collective results showed that fucoidan could restore autophagic flux and has potential value in attenuating the formation and destabilization of carotid atherosclerotic plaques. However, a recent study described that fucoidan inhibited autophagy, which is counter to our results [44]. A possible explanation for this difference may be that the regulation of autophagy in the pathogenesis of different diseases is complex. For example, in hepatic injury, fucoidan inhibits the phosphorylation of JAK2 and STAT1, which then blocks the degradation of p62 and reduces autophagy [44]. While in human gastric carcinoma AGS cells, fucoidan concomitantly causes autophagic cell death by the upregulation of beclin1 and the conversion of LC3I to LC3II [45].

Our results also showed that the protective effects of fucoidan were enhanced by treatment with the autophagy agonist Rapa and alleviated by treatment with the autophagy inhibitor 3-MA. These findings indicate the participation of autophagy in NLRP3 inflammasome activity in the pathogenesis of atherosclerosis. Next, we examined how fucoidan-induced autophagy regulated the NLRP3 inflammasome. It is important to note that p62/SQSTM1, known as a selective autophagy substrate, is a multidomain adaptor protein that localizes at the autophagosome membrane [46]. In autophagy, p62 sequesters K63-linked polyubiquitinated proteins to the autophagic machinery for degradation through its ubiquitin-association and LC3-interacting domains [47, 48]. Recent evidence of p62-mediated clearance of the NLRP3 inflammasome has been described. Our team recently demonstrated that the scaffold adaptor protein p62 can recognize and transport ubiquitinated NLRP3 to autophagic vesicles for selective degradation in oxLDL stimulated foam cells [31]. In the present study, foam cells treated with fucoidan displayed decreased expression of the NLRP3 inflammasome and p62/SQSTM1 accumulation. In addition, fucoidan promoted the colocalization of NLRP3 and p62. To illuminate this complicated relationship, we transfected p62-siRNA to reduce the expression of p62. The p62-siRNA significantly attenuated the inhibitory effect of fucoidan on the expression of NLRP3, ASC, caspase-1, and the downstream inflammatory factor IL-1*β*. Thus, after diminishing p62 expression by siRNA, the anti-inflammatory effect of fucoidan was eliminated. Furthermore, when the expression of ATG5 was reduced, autophagy triggered by fucoidan was suppressed, and the anti-inflammatory effect of fucoidan was decreased. These data indicate that the administration of fucoidan induces p62-dependent selective autophagy of the NLRP3 inflammasome in foam cells, and thus having an anti-inflammatory effect.

In conclusion, our study demonstrated the atheroprotective effects of fucoidan and provided the first data of the novel underlying mechanisms. Fucoidan could inhibit NLRP3 inflammasome activation by enhancing p62/SQSTM1-dependent selective autophagy to alleviate atherosclerosis. These novel results implicated fucoidan as a valuable candidate drug for antiatherosclerosis therapy and provided a molecular basis for its clinical application in atherosclerosis treatment.

## Data Availability

The data used to support the findings of this study are available from the corresponding author upon request.

## Supplementary Materials

Supplementary materials The chemical structure of fucoidan purified from Fucus vesiculosus. Fucoidan alleviates atherosclerosis via enhancing selective autophagy and inhibiting NLRP3 inflammasome activation.
