# Supplementary material for: Fucoidan Inhibits NLRP3 Inflammasome Activation by Enhancing p62/SQSTM1-Dependent Selective Autophagy to Alleviate Atherosclerosis
Source: Oxid Med Cell Longev. 2020 Aug 6;2020:3186306. doi: 10.1155/2020/3186306 (PMC7812546; doi:10.1155/2020/3186306)
Supplement: Supplementary materials — The chemical structure of fucoidan purified from Fucus vesiculosus. Fucoidan alleviates atherosclerosis via enhancing selective autophagy and inhibiting NLRP3 inflammasome activation. [file 3186306.f1.pdf]

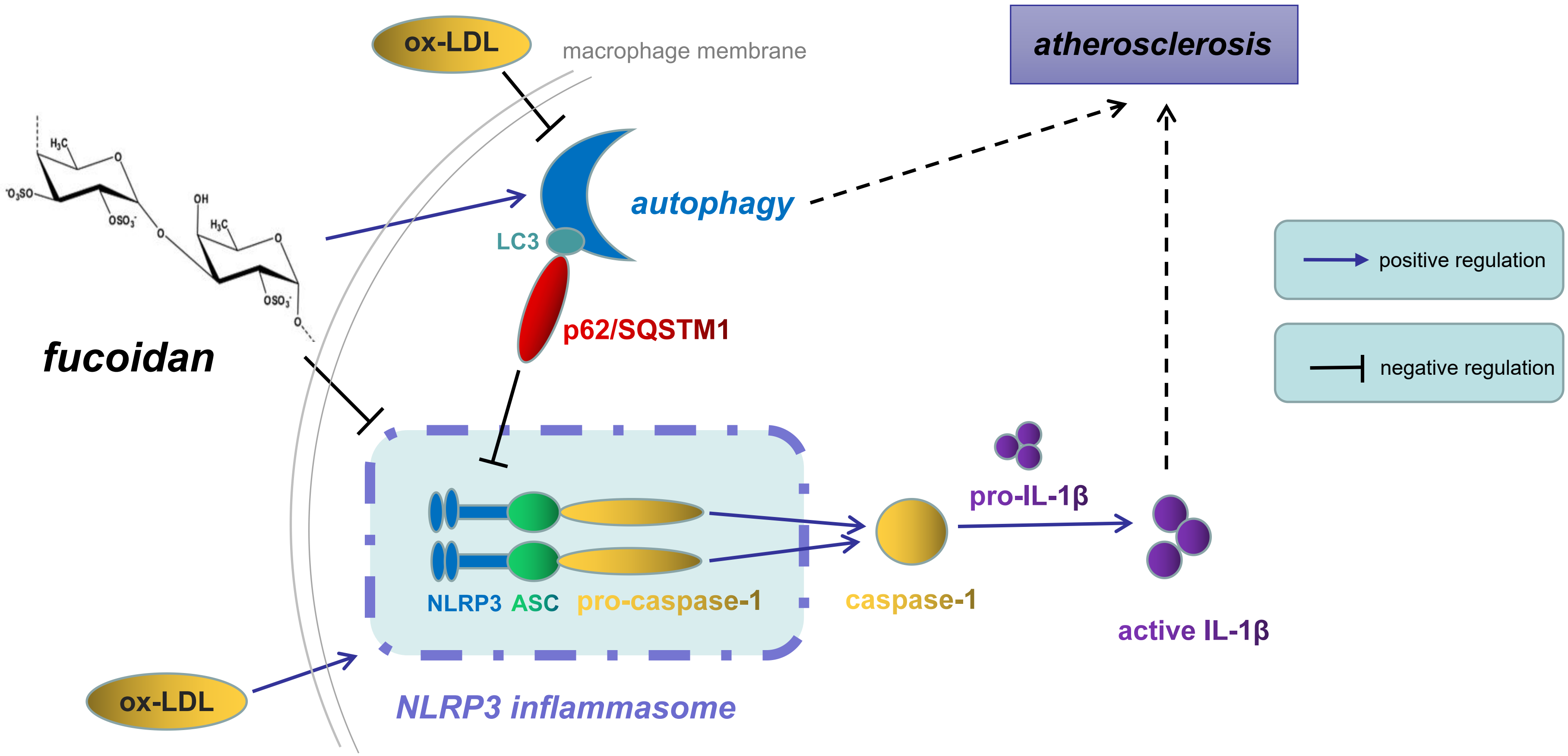

The chemical structure of fucoidan purified from *Fucus vesiculosus.*Fucoidan alleviates atherosclerosis via enhancing selective autophagy and inhibiting NLRP3 inflammasome activation.
